# Diagnostic limitations and procedure-related implantation in follicular-patterned thyroid tumors: a focused review with two illustrative cases

**DOI:** 10.3389/fendo.2026.1927184

**Published:** 2026-10-21

**Authors:** Wenjie Zhang, Yuanyuan Chen, Jie Xiao, Ying Liu, Pin Wang, Hong Liu, Jiechao Ren, Jian Wu

**Affiliations:** The Center of Breast and Thyroid Surgery, Department of General Surgery, The Affiliated Hospital of Southwest Jiaotong University, The Third People’s Hospital of Chengdu, Chengdu, Sichuan, China

**Keywords:** Bethesda system, DICER1, follicular-patterned thyroid tumor, needle-tract implantation, tunnel implantation

## Abstract

The rapid adoption of thyroid radiofrequency ablation (RFA) and remote-access thyroid surgery has changed the therapeutic landscape for thyroid nodules. These approaches can reduce visible scarring, shorten recovery, and offer alternatives to conventional surgery in appropriately selected patients. However, their expansion has also exposed a persistent diagnostic vulnerability: follicular-patterned thyroid tumors cannot be definitively classified as benign or malignant by cytology alone, because the diagnosis of follicular thyroid carcinoma requires histologic assessment of capsular and/or vascular invasion. This review first summarizes the current clinical context of minimally invasive thyroid intervention, the diagnostic boundaries of fine-needle aspiration and Bethesda reporting in follicular-lineage lesions, the definitions and published evidence for needle-tract or surgical-tunnel implantation, and the molecular context of DICER1-altered follicular-patterned tumors. Two implantation-related cases are used as illustrative clinical scenarios rather than as the primary evidentiary basis. The Discussion then addresses the major controversies, including procedural expansion, the role of benign cytology as a preprocedural gatekeeper, the uncertain role of molecular testing before ablation, terminology, patient selection, surveillance, research gaps, and potential developments. The available literature remains dominated by case reports and small series, and causal inference is limited. Nevertheless, repeated reports of tract- or tunnel-aligned deposits in follicular-lineage tumors suggest that implantation risk should be considered when selecting patients for ablative or remote-access interventions. Molecular alterations such as DICER1 RNase IIIb hotspot mutations may provide biological context, but they should not replace histologic classification. Future progress will require standardized reporting, prospective registries, molecularly annotated cohorts, and preprocedural algorithms that integrate imaging, cytology, molecular testing, procedural anatomy, and patient preference.

## 1 Introduction

Minimally invasive thyroid interventions have moved from highly specialized procedures into routine discussions of thyroid nodule management. Thermal ablation, particularly RFA, is now used for selected benign thyroid nodules and recurrent thyroid cancers, while remote-access endoscopic and robotic thyroidectomy techniques are increasingly considered by patients who prioritize cosmetic outcomes. In carefully selected settings, these techniques can reduce visible cervical scarring and preserve quality of life (1–3). Their success, however, depends on a premise that is sometimes difficult to satisfy: the lesion being treated must be biologically appropriate for the chosen procedure.

Follicular-patterned thyroid tumors expose the weak point in that premise. The diagnostic distinction between follicular adenoma and follicular thyroid carcinoma is not based on cytologic atypia alone; it requires demonstration of capsular and/or vascular invasion in a surgical specimen (4, 5). FNA can detect suspicious nuclear features in many papillary thyroid carcinomas and can categorize nodules according to Bethesda risk strata, but it cannot reliably exclude invasive follicular carcinoma when the decisive evidence is architectural and capsular. This limitation is not a technical failure of the cytopathologist; it is a conceptual boundary of the diagnostic method. The issue becomes clinically important when a nodule with benign or equivocal cytology is directed toward ablation or extensive minimally invasive manipulation rather than diagnostic excision. If an apparently benign follicular-patterned tumor is actually invasive, delayed diagnosis, incomplete local control, and unusual recurrence patterns may follow. Among the most unsettling of these patterns is procedure-related implantation, in which tumor deposits appear along a prior needle tract, ablation route, endoscopic tunnel, or remote-access corridor (6–14).

Procedure-related implantation is rare, and its rarity has encouraged two opposing interpretations. One school of thought treats it as an exceptional technical complication that should not alter the general enthusiasm for minimally invasive techniques. Another regards it as a sentinel event: uncommon but revealing, because it exposes the interaction between diagnostic uncertainty, tumor architecture, mechanical displacement, and tissue-plane creation. A third, emerging view asks whether molecular subsets of follicular-patterned tumors may be more likely to behave in a biologically active way despite bland cytology. This review evaluates these positions with emphasis on balanced clinical interpretation. We therefore recast two implantation-related cases as illustrative examples within a focused review. The aim is to clarify the boundary conditions under which these procedures may become problematic, especially when follicular-lineage neoplasia has not been adequately excluded before intervention.

This article is designed as a focused narrative review with illustrative cases. It synthesizes current concepts relevant to follicular-patterned thyroid tumors, cytologic limitations, minimally invasive thyroid procedures, and tract- or tunnel-related implantation. The objective is to provide a state-of-the-art overview and conceptual framework rather than a pooled estimate of incidence. The literature search strategy was built around combinations of the following terms: follicular thyroid carcinoma, follicular-patterned thyroid tumor, follicular neoplasm, fine-needle aspiration, needle-tract implantation, needle-tract seeding, tumor seeding, radiofrequency ablation, endoscopic thyroidectomy, robotic thyroidectomy, tunnel implantation, and DICER1 mutation. Searches of PubMed, Embase, and Web of Science were performed through December 2025. Eligible reports included implantation or atypical tract/tunnel recurrence involving follicular thyroid carcinoma, follicular neoplasm, follicular variant papillary thyroid carcinoma, follicular tumors of uncertain malignant potential, poorly differentiated thyroid carcinoma with follicular lineage, or related follicular-patterned entities. Reports limited to classical papillary thyroid carcinoma were not used as core evidence, although they were considered for general mechanistic context. Because implantation events are rare, the evidence base consists mostly of case reports, small case series, retrospective observations, and mechanistic reasoning. The review therefore emphasizes patterns, plausibility, terminology, and research gaps rather than definitive causation.

## 2 Clinical context: minimally invasive thyroid intervention and the follicular cytology gap

### 2.1 Expansion of RFA and remote-access thyroid surgery

RFA and other image-guided ablation techniques are now established options for carefully selected benign thyroid nodules and selected recurrent thyroid cancers (1, 2). The modern ablation paradigm is built on three assumptions: the target lesion has been appropriately characterized, the procedure can achieve effective volume reduction or tumor control, and the risk profile is acceptable compared with surgery. These assumptions are usually reasonable for clearly benign nodules, but they become less secure when the lesion has a follicular architecture that cytology cannot fully classify. Remote-access endoscopic and robotic thyroid procedures have a different rationale (3). They are surgical rather than ablative, but they create long subcutaneous or intermuscular working spaces that do not exist in conventional open thyroidectomy. These approaches can be attractive for selected patients, yet they depend on careful patient selection, appropriate tumor size and risk profile, surgeon experience, and a clear understanding of oncologic trade-offs. The absence of a neck scar is not equivalent to oncologic neutrality; the route of access becomes part of the oncologic environment. These modalities place substantial weight on preprocedural characterization. For clearly benign nodules, ablation is an accepted option in selected patients; for selected low-risk malignant disease, remote-access approaches may be considered in experienced centers. For lesions with possible follicular-lineage biology, however, the preprocedural status is more complex because definitive classification may depend on histologic assessment that is not available before ablation.

### 2.2 The follicular cytology gap

The diagnostic problem begins with the definition of follicular thyroid carcinoma (4). Unlike classical papillary thyroid carcinoma, which is often diagnosed by characteristic nuclear features, follicular thyroid carcinoma is defined by invasion. The decisive findings are capsular invasion, vascular invasion, or both. These features require a properly oriented histologic specimen with the tumor-capsule interface and vessels available for examination. Cytology samples cells, not the complete capsule. This distinction explains why a benign or nonmalignant FNA result cannot always exclude a clinically relevant follicular-lineage neoplasm. A macrofollicular tumor with abundant colloid and bland cells can appear reassuring on cytology, while the same lesion may later prove to have capsular invasion on surgical pathology. Conversely, overcalling every follicular-patterned nodule as high risk would expose many patients with benign adenomas to unnecessary surgery. The problem is not simply sensitivity or specificity; it is that the test and the disease definition operate at different levels of tissue organization.

Bethesda reporting improves communication of cytologic risk, but it does not eliminate this boundary (5). Bethesda II is expected to have a low malignancy risk in ordinary practice, but a follicular carcinoma with bland cytology can still enter this category if the sampled material resembles a benign hyperplastic or adenomatous nodule. Indeterminate categories such as follicular neoplasm/suspicious for follicular neoplasm more clearly signal the need for histologic evaluation, but the most challenging cases are those in which the possibility of follicular neoplasia is not recognized before ablation. This diagnostic boundary is relevant to procedural planning. Ablation alters tissue and may complicate later histologic interpretation, whereas remote-access or endoscopic surgery creates access planes that differ from those used in conventional open thyroidectomy. For follicular-patterned lesions, preprocedural evaluation therefore must establish not only whether the nodule appears low risk, but also whether the available diagnostic information is adequate for a tissue-destructive or extensively manipulative procedure.

## 3 Procedure-related implantation in follicular-patterned thyroid tumors

### 3.1 Definitions and diagnostic criteria

Procedure-related implantation refers to tumor deposits that arise along a prior procedural pathway and are anatomically difficult to explain by ordinary lymphatic or hematogenous dissemination alone. In the thyroid setting, the relevant pathways include FNA or core-needle tracts, RFA needle trajectories, endoscopic surgical tunnels, robotic access corridors, and chest-wall or axillary entry routes (6–14). The term should be used carefully: not every local recurrence is implantation, and not every subcutaneous tumor deposit near a scar proves seeding. A diagnosis of implantation is strongest when several features converge: spatial alignment between the deposit and a documented procedural path; occurrence in tissue that does not normally contain thyroid tissue or intrinsic lymphoid architecture, such as subcutaneous tissue, strap muscle, or chest-wall soft tissue; a plausible interval between the procedure and detection; histologic or immunophenotypic confirmation of thyroid origin; and careful consideration of alternative explanations such as residual thyroid bed disease, nodal metastasis, or distant cutaneous metastasis.

Experimental and clinicopathologic explanations for implantation have focused on viable tumor cell displacement, survival within a newly created tissue environment, and subsequent growth. Needle passage has been proposed to drag cells along a tract (8–10, 22, 23). In image-guided ablation, tissue disruption, thermal injury, intranodular pressure changes, and post-ablation remodeling may alter the local environment. In endoscopic and robotic surgery, broad subcutaneous or intermuscular working spaces and repeated instrument passage create anatomical routes that differ from conventional open thyroidectomy. Published reports have proposed several characteristics that may be relevant in follicular-patterned tumors, including encapsulation, capsular disruption, follicular architecture, reduced cellular cohesion, and capsular or vascular invasion. The available literature remains descriptive, and denominator data are limited; therefore, these characteristics should be regarded as reported associations or biologically plausible factors rather than validated predictors of implantation.

### 3.2 Published evidence from FNA, RFA, and endoscopic/robotic surgery

The published literature on tract- and tunnel-related implantation in follicular-patterned thyroid tumors is small but instructive (6–14). The reported events are summarized in **Table 1**. Reports include needle-tract implantation after FNA of follicular neoplasm or follicular carcinoma, subcutaneous deposits after biopsy, and recurrence along endoscopic or robotic access corridors. Some reports provide convincing anatomic and histologic evidence, whereas others are best interpreted as strongly suggestive because procedural details or imaging documentation are incomplete. Several patterns recur. Implantation can be detected within months, but delayed presentation after years has also been described. Deposits may occur in subcutaneous tissue, strap muscle, muscular planes, chest-wall access sites, or along the sternocleidomastoid region. Management usually involves surgical excision of the implant, completion thyroidectomy or reoperation when indicated, lymph node dissection if nodal disease is present, and radioactive iodine in selected cases. Outcomes vary, and the rarity of events prevents robust outcome modeling. Across the published cases, the available data provide a descriptive map of procedural settings, histologic subtypes, time intervals, and anatomic sites. The strongest reports combine anatomic alignment with the procedural route, thyroid-origin confirmation, and exclusion of lymph node tissue or ordinary thyroid-bed recurrence.

**Table 1:** Reported tract- or tunnel-related implantation events in follicular-patterned thyroid tumors.

Author (year)	Procedure category	Follicular-lineage histology	Time to implantation	Implantation site	Interpretive note
Ito et al. (2006) (6)	FNA needle tract	Follicular carcinoma	Weeks to months after FNA	Subcutaneous tissue along the needle tract	Definite needle-tract implantation
Panunzi et al. (1994) (12)	FNA needle tract	Follicular neoplasm/carcinoma	Approximately 8 months	Subcutaneous needle tract	Strongly inferred implantation
Uchida et al. (2007) (7)	FNA needle tract	Follicular thyroid carcinoma	Delayed after FNA	Needle track dissemination	Case report supporting plausibility
Hayashi et al. (2020) (9)	FNA needle tract	FTC and PDTC in a subset	Variable	Along FNAB tract	Retrospective cohort; follicular lineage overrepresented among events
Beninato et al. (2012) (11)	Endoscopic transaxillary tunnel	Follicular thyroid neoplasm	Not reported	Surgical tunnel	Definite tunnel-related recurrence
Chabrillac et al. (2020) (13)	Robotic transaxillary tunnel	FTC with oncocytic features	Delayed, years	Muscular/subcutaneous tunnel	Strongly inferred tunnel implantation
Zhang et al. (2024) (14)	Endoscopic and traditional thyroid surgery	FTC and FVPTC	8–60 months	Subcutaneous tunnel, chest wall, sternocleidomastoid region	Definite or highly plausible implantation in a case series

FNA, fine-needle aspiration; FTC, follicular thyroid carcinoma; FVPTC, follicular variant papillary thyroid carcinoma; PDTC, poorly differentiated thyroid carcinoma.

### 3.3 Illustrative clinical scenarios

#### 3.3.1 Case 1: cytologically benign nodule later diagnosed as follicular thyroid carcinoma with post-RFA implantation

A 35-year-old man presented with a right thyroid nodule detected on routine ultrasonography. The imaging and intraoperative findings are presented in **Figures 1**, **2**. The initial ultrasound showed a well-defined hypoechoic nodule without microcalcifications or suspicious lymphadenopathy. Ultrasound-guided FNA was interpreted as Bethesda II, consistent with adenomatous goiter. No follicular neoplasm was suspected before treatment, and the lesion was managed as a presumed benign thyroid nodule with RFA rather than diagnostic surgical excision. Less than two years after ablation, follow-up ultrasonography revealed a new anterior cervical subcutaneous soft-tissue nodule located superficially to the strap muscles and spatially corresponding to the ablation needle trajectory. Multiple enlarged central compartment and right lateral neck lymph nodes were also identified. FNA of a right level V lymph node showed thyroid follicular epithelial cells, raising suspicion for metastatic follicular thyroid neoplasm.

**Figure 1:**
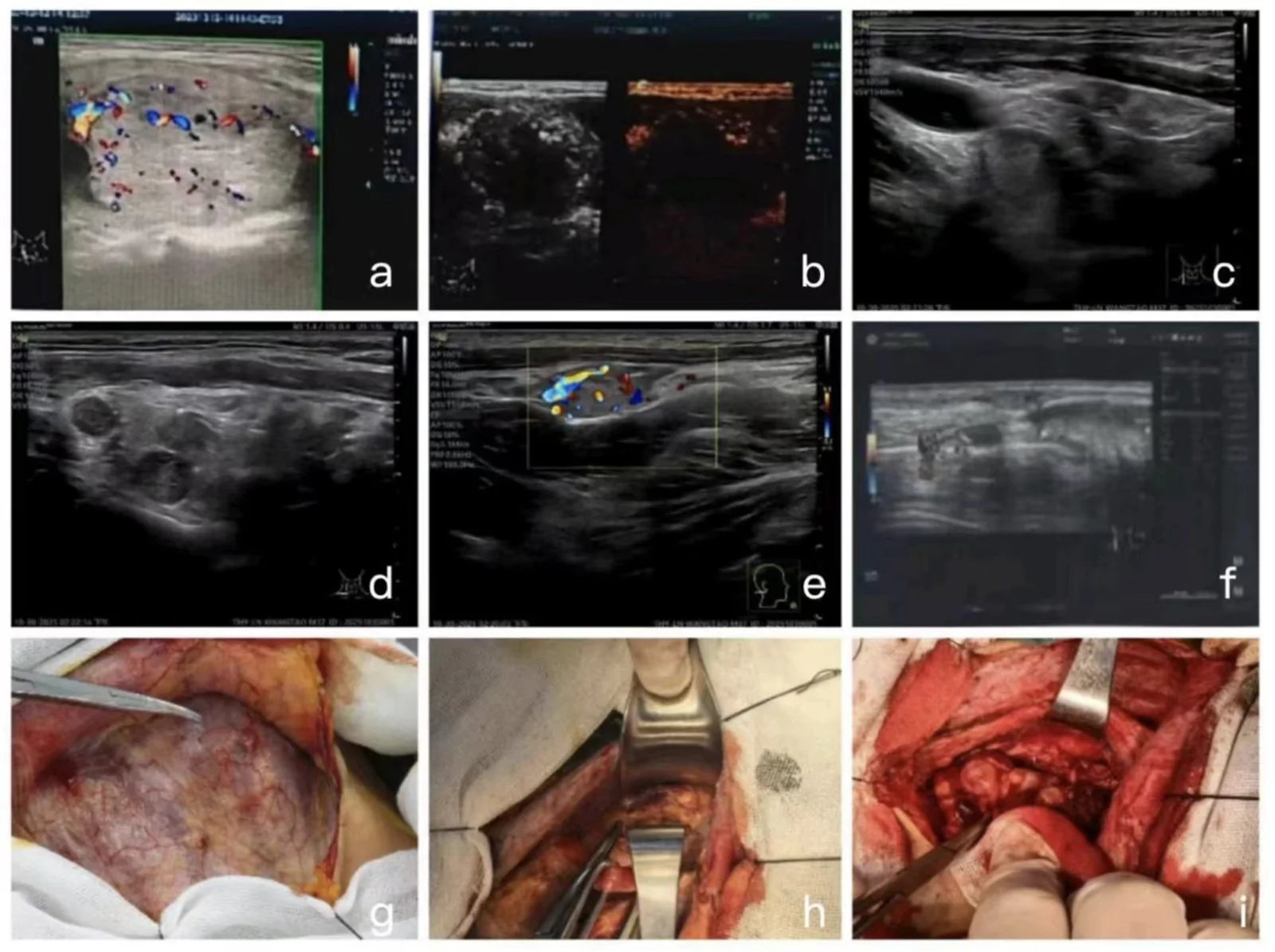
Imaging and intraoperative findings in Case 1. **(a)** Pre-ablation ultrasonographic image of the right thyroid nodule. **(b)** Post-ablation contrast-enhanced ultrasonographic image. **(c, d)** Post-ablation recurrent lesion in the thyroid operative field. **(e)** Enlarged level V lymph node. **(f)** Metastatic lesion on the anterior cervical strap muscles, consistent with needle-tract implantation. **(g–i)** Intraoperative photographs demonstrating a strap-muscle implant, lateral neck nodal disease, and recurrent tumor in the thyroid operative field.

**Figure 2:**
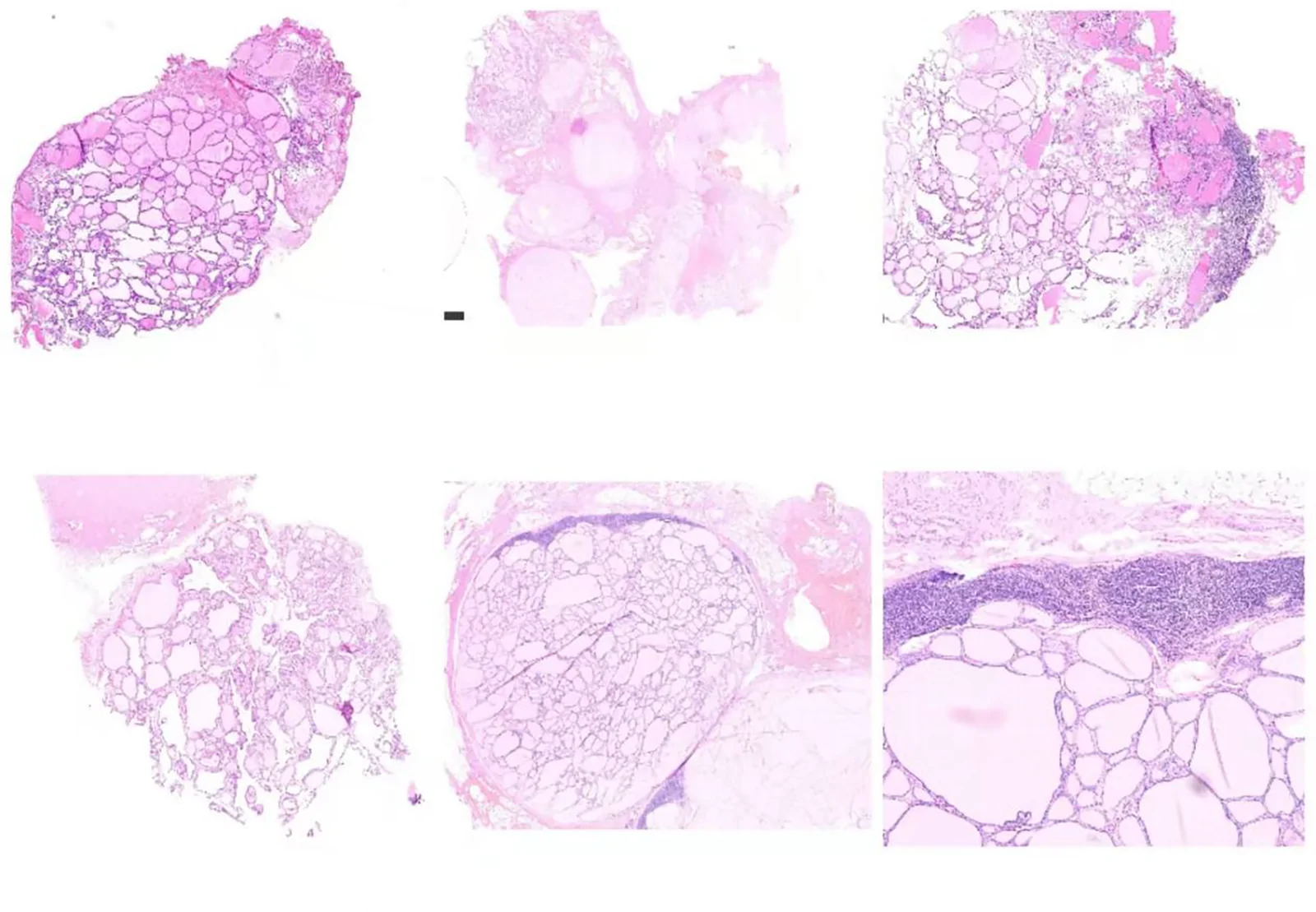
Histologic features in Case 1. The tumor was well circumscribed by a thick fibrous capsule and showed predominantly macrofollicular architecture with focal papillary formations and abundant colloid. Capsular invasion was identified. Panels show the needle-tract implantation lesion, post-ablation thyroid lesion, and metastatic lymph nodes in pretracheal, central, and lateral cervical compartments.

The patient underwent total thyroidectomy with central compartment lymph node dissection and selective right lateral neck dissection. Histopathology demonstrated multifocal follicular thyroid carcinoma predominantly involving the right lobe, definite capsular invasion, central and pretracheal nodal metastases, and a follicular carcinoma deposit in anterior cervical subcutaneous tissue and strap muscle. The soft-tissue deposit lacked lymphoid architecture and was aligned with the prior RFA needle path, supporting needle-tract implantation. Targeted next-generation sequencing detected a DICER1 c.5428G>C (p.D1810H) RNase IIIb hotspot mutation with a variant allele frequency of 33.88%. Rare KIT p.W359R and PIK3CA p.P104R variants were also detected and classified as variants of uncertain clinical significance. The molecular findings support a follicular-patterned tumor background.

#### 3.3.2 Case 2: tunnel-related implantation after endoscopic thyroid surgery

A 31-year-old man previously underwent endoscopic partial thyroidectomy for a right-lobe follicular-patterned thyroid nodule. The clinical, imaging, and histopathologic findings are presented in **Figure 3**. The initial postoperative course was uneventful. Several years later, he developed recurrent thyroid bed disease, multiple implantation deposits along the endoscopic tunnel tract, and additional anterior chest-wall nodules. Histopathology confirmed recurrent follicular-patterned thyroid carcinoma involving the surgical cavity, soft tissue, and tunnel pathway. No distant metastasis was identified at diagnosis. The implantation sites corresponded anatomically to the subcutaneous tunnel created during endoscopic access and to the extracervical entry route near the chest wall, supporting mechanical displacement during endoscopic manipulation. This case illustrates a different procedural pathway but the same conceptual problem: a follicular-patterned tumor with invasive potential was exposed to a minimally invasive access route before the full biologic significance of the lesion was appreciated.

**Figure 3:**
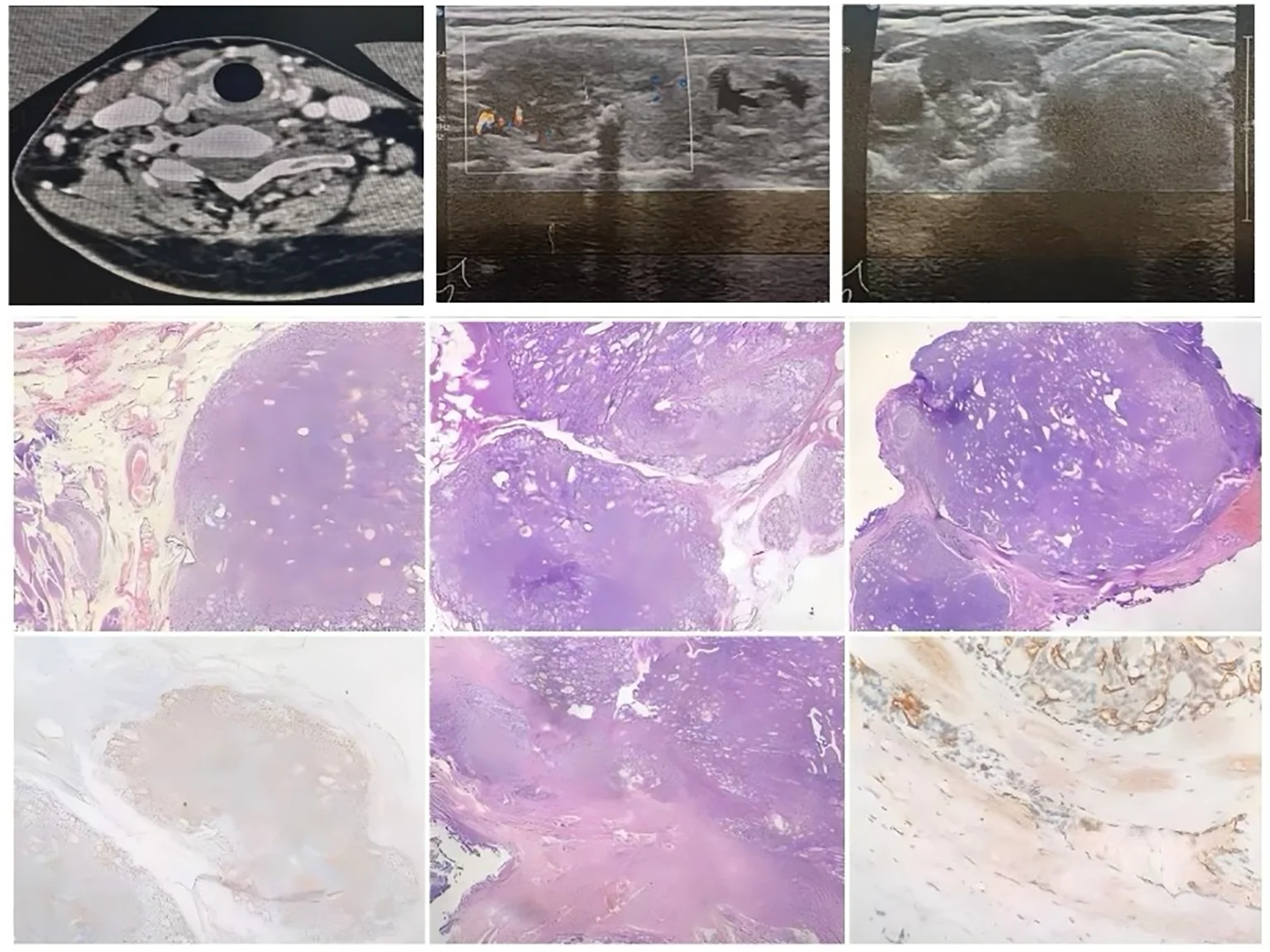
Clinical, imaging, and histopathologic findings in Case 2. Physical examination and imaging demonstrated subcutaneous and thyroid bed lesions. Histology of the recurrent thyroid lesion showed a follicular tumor with capsular and vascular invasion, and immunohistochemistry supported thyroid origin in the subcutaneous implant.

## 4 Biologic and molecular context: why some follicular tumors may be underestimated

DICER1 alterations have become increasingly recognized in follicular-patterned thyroid tumors (15–21). The molecular and clinicopathologic themes are summarized in **Table 2**. RNase IIIb hotspot mutations involving codons such as D1709 and D1810-E1813 are reported across pediatric, adolescent, young adult, and adult thyroid tumors with follicular architecture (15–21). These tumors may show macrofollicular growth, encapsulation, bland cytologic features, infarction-prone morphology, or low-grade appearance, yet a subset demonstrates capsular invasion, vascular invasion, recurrence, or progression. Reported DICER1-altered follicular-patterned tumors illustrate a biological-diagnostic discordance: bland cytology and encapsulated architecture may coexist with capsular invasion, vascular invasion, recurrence, or progression in a subset of cases (15–21). At the same time, many reported lesions remain low grade. Current studies therefore describe a spectrum of behavior rather than a uniform aggressive phenotype. In the first illustrative case, DICER1 p.D1810H is included as molecular context for a follicular-patterned tumor that was initially treated as benign and later showed invasive and metastatic behavior. Published evidence has not established a direct causal relationship between DICER1 alteration and procedure-related implantation; the association remains hypothesis-generating.

**Table 2:** DICER1-altered follicular-patterned thyroid tumors: selected molecular and clinicopathologic themes.

Theme	Current evidence	Relevance to current evidence
Mutation pattern	RNase IIIb hotspot variants have been reported at D1709 and the D1810-E1813 region, often as somatic events or second hits in susceptible backgrounds.	Reported in follicular-patterned tumors, especially when cytology and imaging are discordant.
Morphology	Reported tumors often show follicular, macrofollicular, encapsulated, or deceptively bland features; infarction-prone histology has also been described.	These morphologic patterns help explain why some lesions may appear low risk before definitive histology.
Clinical behavior	Most reported lesions are low grade, but subsets show capsular invasion, vascular invasion, nodal disease, recurrence, or high-grade progression.	Reported behavior is heterogeneous; no stand-alone management rule has been validated for DICER1 status.
Relation to implantation	Direct evidence linking DICER1 to implantation metastasis is lacking; the present case provides a biologically plausible but hypothesis-generating association.	Current evidence supports contextual rather than causal interpretation of the association.
Available evidence	Published studies are heterogeneous and rarely include procedural details relevant to ablation or remote-access surgery.	The literature remains insufficient for genotype-based procedural recommendations.

## 5 Current evidence relevant to preprocedural assessment and follow-up

Current evidence relevant to preprocedural assessment comes from practice guidelines, Bethesda-based cytologic risk stratification, reported implantation cases, and small molecularly annotated series. The literature does not provide a validated risk score for implantation, but it identifies recurring domains that are relevant when follicular-patterned nodules are considered for ablation or remote-access surgery.

These domains are summarized descriptively in **Table 3**. The table is not intended as a management algorithm; rather, it organizes the forms of evidence currently used or reported in relation to diagnostic certainty, procedural route, molecular context, and postprocedural follow-up.

**Table 3:** Current evidence domains relevant to preprocedural assessment and follow-up in follicular-patterned thyroid nodules considered for ablation or remote-access surgery.

Evidence domain	Current evidence or observation	Relevance to interpretation
Cytology	Bethesda reporting improves communication of malignancy risk, but cytology cannot assess capsular or vascular invasion in follicular-patterned tumors (4, 5).	Benign or nonmalignant cytology is less definitive when follicular-lineage neoplasia remains possible.
Imaging-cytology concordance	Ablation guidelines emphasize careful selection of benign nodules, while reported cases show that deceptively low-risk nodules may later prove invasive (1, 2, 6–14).	Discordance among growth, size, sonographic pattern, and cytology is a recurring source of preprocedural uncertainty.
Procedural route	Reported implantation deposits have occurred along FNA tracts, ablation paths, endoscopic tunnels, robotic access corridors, and chest-wall or axillary routes (6–14).	Route documentation is necessary for distinguishing tract/tunnel implantation from nodal disease, thyroid-bed recurrence, or distant cutaneous metastasis.
Molecular context	DICER1 and other follicular-patterned molecular alterations have been reported in tumors with bland morphology and variable clinical behavior (15–21).	Molecular findings may add context, but no genotype is currently validated as a predictor of implantation.
Follow-up	Published cases describe implantation detected from months to years after the index procedure, and follow-up protocols vary across reports (6–14).	Assessment of the prior procedural route may be relevant when new subcutaneous, muscular, chest-wall, or tunnel-aligned lesions are detected.

## 6 Discussion

This review highlights a central tension in contemporary thyroid practice: minimally invasive thyroid procedures are increasingly refined and clinically valuable, yet their safe application depends on the accuracy of preprocedural tumor classification. For follicular-patterned thyroid tumors, this requirement is inherently difficult to satisfy because the distinction between benign and malignant disease depends on capsular and/or vascular invasion, which cannot be fully assessed by cytology. The issue is therefore not simply whether FNA performs well in general thyroid nodule evaluation, but whether cytology can provide the type of information required before tissue-destructive or route-creating interventions are selected. In this context, follicular-patterned lesions represent a diagnostic boundary condition for ablation and remote-access surgery.

The available evidence suggests that procedure-related implantation should not be dismissed as an isolated technical curiosity. Reported events remain rare, but their repeated occurrence along needle tracts, ablation routes, endoscopic tunnels, and remote-access corridors indicates a recognizable pattern. These events are best understood as the result of interaction between tumor biology, procedural manipulation, and diagnostic uncertainty. A thyroid tumor with follicular architecture may appear cytologically bland, yet still possess invasive potential. When such a lesion undergoes ablation or extensive minimally invasive manipulation before definitive histologic classification, viable tumor cells may be displaced into tissue planes that would not otherwise be exposed to tumor. This interpretation does not imply that minimally invasive procedures are broadly unsafe; rather, it identifies a specific setting in which the margin for preprocedural diagnostic error is narrow.

The two illustrative cases in this review support this concept. In the RFA-associated case, a nodule interpreted as Bethesda II was treated as benign, but subsequent pathology demonstrated follicular thyroid carcinoma with capsular invasion, nodal metastases, and a soft-tissue implant aligned with the prior ablation tract. This sequence emphasizes how a reassuring cytologic result can become clinically misleading when the underlying lesion is follicular-lineage and the planned treatment reduces the opportunity for later histologic assessment. The endoscopic case illustrates a parallel mechanism through a different procedural route: creation of a surgical tunnel may provide an anatomic pathway for tumor displacement when a follicular-patterned tumor with invasive potential is manipulated before its biologic behavior is fully recognized. Together, these cases suggest that implantation is not only a procedural event, but also a marker of preprocedural diagnostic insufficiency.

A major challenge is that current literature cannot define the true incidence or individual-level risk of implantation. Most available evidence consists of case reports, small case series, and retrospective observations, often without clear denominators. As a result, the field risks two opposite errors. One is overinterpretation, in which rare implantation events are used to argue against otherwise appropriate minimally invasive treatment. The other is underinterpretation, in which rarity is taken to mean irrelevance. A balanced view is that implantation events are uncommon but informative. They do not justify abandoning RFA or remote-access surgery, but they do justify more careful attention to patient selection, procedural documentation, and follow-up of the access route.

The molecular findings should also be interpreted within this balanced framework. DICER1 alterations, particularly RNase IIIb hotspot mutations, have expanded understanding of follicular-patterned thyroid tumor biology. These tumors may show bland cytologic or architectural features while retaining the potential for invasion, recurrence, or progression in a subset of cases. In the present RFA-associated case, the DICER1 p.D1810H mutation provides biologic context for a tumor that was initially underestimated. However, the evidence does not establish DICER1 as a direct cause of implantation or as a validated predictor of procedure-related seeding. Its main significance here is conceptual: molecular alterations may help explain why some follicular-patterned tumors appear low risk before treatment, but they cannot replace histologic assessment of invasion.

These observations have practical implications for how clinicians interpret benign or indeterminate findings before minimally invasive intervention. A benign cytologic diagnosis should be weighed against the entire clinicoradiologic picture, especially in solid, enlarging, encapsulated, or otherwise follicular-patterned nodules. When imaging, cytology, clinical behavior, or molecular results are discordant, diagnostic surgery may provide greater oncologic certainty than ablation. Similarly, remote-access approaches should be considered not only in terms of cosmetic benefit, but also in terms of the additional tissue planes and access routes created during the operation. The key clinical question is not whether a minimally invasive approach is technically feasible, but whether the lesion has been characterized well enough for that approach to be oncologically appropriate.

The discussion also underscores the need for clearer terminology. Terms such as implantation, seeding, tract recurrence, tunnel recurrence, and subcutaneous metastasis are sometimes used inconsistently. Future reports should distinguish definite implantation, supported by tract alignment and histologic confirmation, from probable or possible implantation when documentation is incomplete. Such standardization would improve literature synthesis and prevent both exaggeration and minimization of risk. Equally important is the systematic recording of procedural details, including needle path, number of passes, ablation parameters, hydrodissection, tumor capsule status, access route, specimen handling, and the exact anatomic distribution of recurrence.

Several limitations should be acknowledged. The illustrative cases cannot establish causality, and the rarity of implantation limits the strength of inference. The literature is heterogeneous and subject to publication bias, because unusual complications are more likely to be reported than uneventful procedures. Molecular testing was available only in one case, and the relationship between DICER1, other follicular-lineage molecular profiles, and implantation risk remains hypothesis-generating. Therefore, the conclusions of this review should be regarded as practice-informing.

In conclusion, follicular-patterned thyroid tumors remain a critical challenge in the era of thyroid ablation and remote-access surgery. The central issue is not whether minimally invasive thyroid procedures should be restricted broadly, but whether diagnostic uncertainty in follicular-lineage lesions is being sufficiently recognized before these procedures are selected. Procedure-related implantation is rare, but it exposes an important vulnerability at the intersection of cytology, tumor biology, and procedural anatomy. Until prospective, molecularly annotated, and procedure-specific data become available, histologic assessment of capsular and vascular invasion should remain central when follicular carcinoma cannot be confidently excluded.

## Data Availability

The raw data supporting the conclusions of this article will be made available by the authors, without undue reservation.
